# Improving the immunomodulatory function of mesenchymal stem cells by defined chemical approach

**DOI:** 10.3389/fimmu.2022.1005426

**Published:** 2022-09-20

**Authors:** Jintao Cheng, Yuan Feng, Xiao Feng, Donghao Wu, Xu Lu, Zhihua Rao, Cuiping Li, Nan Lin, Changchang Jia, Qi Zhang

**Affiliations:** ^1^ Cell-Gene Therapy Translational Medicine Research Center, The Third Affiliated Hospital, Sun Yat-sen University, Guangzhou, China; ^2^ Department of Hepatobiliary Surgery, The Third Affiliated Hospital, Sun Yat-sen University, Guangzhou, China; ^3^ Department of Hepatic Surgery, Liver Transplantation Center, The Third Affiliated Hospital, Sun Yat-sen University, Guangzhou, China; ^4^ Department of Medical Oncology, The Third Affiliated Hospital, Sun Yat-sen University, Guangzhou, China; ^5^ Tangxia Laboratory, The Third Affiliated Hospital, Sun Yat-sen University, Guangzhou, China

**Keywords:** mesenchymal stem cells, small-molecule compounds, immunomodulation, cell therapy, inflammatory cytokines

## Abstract

Mesenchymal stem cell (MSC) is a potential therapeutic material that has self-renewal, multilineage differentiation, and immunomodulation properties. However, the biological function of MSCs may decline due to the influence of donor differences and the *in vitro* expansion environment, which hinders the advancement of MSC-based clinical therapy. Here, we investigated a method for improving the immunomodulatory function of MSCs with the help of small-molecule compounds, A-83-01, CHIR99021, and Y27632 (ACY). The results showed that small-molecule induced MSCs (SM-MSCs) could enhance their immunosuppressive effects on T cells and macrophages. *In vivo* studies showed that, in contrast to control MSCs (Ctrl-MSCs), SM-MSCs could inhibit the inflammatory response in mouse models of delayed hypersensitivity and acute peritonitis more effectively. In addition, SM-MSCs showed the stronger ability to inhibit the infiltration of pro-inflammatory T cells and macrophages. Thus, small-molecule compounds ACY could better promote the immunomodulatory effect of MSCs, indicating it could be a potential improving method in MSC culture.

## Introduction

Since mesenchymal stem cells (MSCs) can secrete a variety of chemokines and immune regulators to affect the physiological microenvironment, they have broad application prospects in the treatment of immune diseases (1–3). Multiple studies have confirmed that MSCs infusion can improve the physiological symptoms of liver ischemia-reperfusion injury, acute-on-chronic liver failure, osteoarthritis, and Crohn’s disease (4–7). MSCs have excellent self-renewal properties. After being isolated from a specific tissue, the number of MSCs required for treatment can be obtained by expanding and culturing MSCs *in vitro* for a certain period of time (8).

The *in vitro* expansion environment of MSCs has always been concerned by scholars in the field. Studies have shown that during the MSC expansion process, various environmental factors such as temperature, radiation, compounds and nutrients can affect the phenotype and biological function of MSCs (8, 9). The immunomodulatory properties of MSCs are their important biological functions. However, MSC-related properties, such as proliferation rate, differentiation potential, and immunomodulatory functions, are prone to significantly decline during cell extraction and culture (10, 11). Therefore, how to maintain the stability of the biological function of MSCs is a big challenge in preclinical research on MSCs.

Several potential approaches have been reported to improve the functional stabilization of MSCs during expansion (8, 12, 13). Through gene editing, the expression or self-renewal capacity of MSC-related functional molecules can be improved. By using embryonic stem cells or induced pluripotent stem cells to expand *in vitro*, they are then induced to differentiate into homogeneous MSCs to ensure the maintenance of their phenotype and function (14–16). However, the method of gene editing is still limited by the stability and safety of the technology, and the method of inducing pluripotent stem cell differentiation is also limited by the current cell culture cost and differentiation efficiency (17–19).

Specific combinations of small molecule compounds were reported contribute to the maintenance of the pluripotency of stem cells and allow for the stable culturing of various types of stem cells (20–22). One of our previous studies indicated that stimulation of MSCs for a certain period of time with the compound cocktail ACY could effectively promote the viability of MSCs and maintain their differentiation potential (23), which demonstrated that ACY is a potential MSC modification material. In this study, we conducted a comprehensive analysis of immunomodulatory biological functions of MSCs against ACY. We evaluated the regulatory effect of ACY-induced MSCs on immune cells represented by T cells and macrophages, and further compared the therapeutic effects of MSCs in mouse models of delayed hypersensitivity and acute peritonitis.

## Materials and methods

### Isolation and culture of MSCs

Following the Clinical Research Ethics Committee of the Third Affiliated Hospital of Sun Yat-sen University and written informed patient consent for tissue collection, MSCs were isolated following the protocol described in the previous study (23). In brief, fat tissues were isolated *via* liposuction from healthy patients defined as no history of malignancy or autoimmune deficiency. The fat tissues were shredded and digested with 0.1% collagenase type I (Sigma-Aldrich) for 30 minutes at 37°C with gentle agitation. The cells were collected and cultured with MSC medium containing low glucose DMEM (Gibco) supplemented with FBS (10%, Gibco), fibroblast growth factor-basic (bFGF, 1 ng/ml, PeproTech) and penicillin/streptomycin (1%, Gibco).

When MSCs were approximately 90% confluent, they would be passaged with a split ratio of 1:3. MSCs of passage 2 were suspended in MSC medium with or without combinations of the following three small molecules, 2μM A-83-01 (MedChemExpress), 3μM CHIR99021 (Selleck), and 2μM Y-27632 (Selleck), and seeded on plates. The medium was changed every 2 days. From passage 5 to passage 7, the MSCs were cultured in MSC medium and used in subsequent analysis.

### Mice

C57BL/6 wild-type mice were purchased from Gempharmatech Co., Ltd. BALB/c wild-type mice were purchased from the SPF (Beijing) Biotechnology Co., Ltd. All animals used for *in vivo* studies were 8-10-week-old male mice that were randomly allocated to each group. All animal protocols were reviewed and approved by the Animal Care and Use Committee of the Third Affiliated Hospital of Sun Yat-sen University.

### Contact hypersensitivity model

Contact hypersensitivity reactions were induced in BALB/c mice as previously described with minor adjustment by 2,4-dinitrofluorobenzene (DNFB) (24). Brief, the reactive solution for pre-sensitization contained 0.5% DNFB (Sigma) in acetone/olive oil (4:1), which was applied to a shaved hind flank once a day for 2 days. 5 days later, all mice were randomly divided into four groups: Sham group, Ctrl group, Ctrl-MSC group and SM-MSC group. 0.25% DNFB solution was used to challenge the right ear of pre-sensitized mice of Ctrl group, Ctrl-MSC group and SM-MSC group, while mice of SM-MSC group or SM-MSC group were intravenously infected with 5 × 10^5^ Ctrl-MSCs or SM-MSCs *via* the tail vein. Ear thickness was measured at 48h post-challenge. In some studies, mice were sacrificed, and ear samples and cervical lymph nodes were harvested for analysis.

### LPS model of endotoxemia

Endotoxemia reactions are induced in BALB/c mice as previously described with minor adjustment by LPS (Sigma) (25). All mice were randomly divided into 3 groups: without MSC group, Ctrl-MSC group and SM-MSC group. All mice were injected i.p. with 2.5μg/g LPS in sterile saline, while mice of Ctrl-MSC group or SM-MSC group were intravenously infected i.p. with 1 × 10^6^ Ctrl-MSCs or SM-MSCs on the day of modeling. After 48h, mice were sacrificed, and peritoneal exudates were collected for flow cytometry analysis by carefully flushing 5 mL of sterile PBS into and out of the peritoneal cavity.

### Flow cytometry

Flow cytometric analyses were performed with Gallios (Beckman Coulter) flow cytometers, and the data were analyzed with the FlowJo (Treestar) software packages.

MSCs were incubated with anti-CD90, anti-CD105, anti-CD73, anti-CD44, anti-CD11B, anti-CD34, anti-CD19, anti-CD45 and anti-HLA-DR antibodies to examine MSC surface markers, which were purchased from BioLegend. Anti-CD3, anti-CD4, anti-CD8, anti-TNF-α and anti-IFN-γ antibodies were used to examine stain T cells. Propidiumiodide (PI; BD) and Annexin V (BD) were used to stain apoptosis cells. Anti-CD86, anti-F4/80 and anti-CD45 antibodies were used to stain macrophages.

### Differentiation analysis

For osteogenic, chondrogenic and adipogenic differentiation, MSCs were stimulated with the Osteogenic Differentiation Kit, Chondrogenic Differentiation Kit and Adipogenic Differentiation Kit, purchased from Cyagen Biosciences as manufacturer’s protocol.

### MSCs/T cells co-cuture assay

MSCs were plated using 24-well plates. For co-culture assays, MSC medium was removed, 2 × 10^6^ CD3^+^ T cells isolated from freshly PBMCs by magnetic bead sorting were plated with or without MSC. T cells were cultured in T cell culture medium containing RPMI 1640 (Gibco) with FBS (10%, Gibco).

For T cell proliferation assay, T cells were activated by anti-CD3 and anti-CD28 (500ng/ml; PeproTech) antibodies. 5,6-carboxyfluorescein diacetatesuccinimidyl ester (CFSE; 5μmol/L; Invitrogen) was used to stain. After 72h of proliferation, T cells were collected and analyzed by flow cytometry.

For T cell apoptosis assay, T cells were activated by anti-CD3 and anti-CD28 (500ng/ml; PeproTech) for 72h. The percentage of apoptotic cells was analyzed by flow cytometry with PI and Annexin V (BD) staining.

For intracellular cytokine staining of T cells, T cells were co-cultured with or without MSCs for 48h. Before analysis, T cells were stimulated for 6h with 50 ng/ml phorbol myristate acetate (PMA, TargetMol), 1 μg/ml ionomycin (StemCells), 3μg/ml brefeldin A (BFA, TargetMol) and 1.4μg/ml monensin (TargetMol). Then,T cells were collected and stained with anti-CD3 antibody before fixation with Fixation Buffer (Biolegend) for 0.5h. After fixation, cells were washed and stained with anti-TNF-α and anti-IFN-γ antibodies in intracellular staining perm wash buffer (Biolegend) for 1h at 4°C.

### MSCs/macrophages co-cuture assay

Macrophages were isolated from bone marrow of 6-8 weeks old C57BL/6 mice, plated on 6-well plates at 2 × 10^5^ cells per well in macrophage media containing RPMI 1640 (Gibco) with heat‐inactivated FBS (10%, Gibco) and 10 ng/ml colony‐stimulating factor (CSF; PeproTech) for 7-10 days. The media were then replaced with media consisting of RPMI 1640 (Gibco) with heat‐inactivated FBS (10%, Gibco) and 100 ng/ml LPS (Sigma‐Aldrich) to induce M1 macrophage differentiation for 24h. MSCs were added to wells at the density of 5 × 10^4^/well and cultured in macrophage media. After 48h, all cells were collected and analyzed by flow cytometry.

### Immunohistochemistry

All of the tissues were fixed in 4% paraformaldehyde (PFA; Sigma), then embedded in paraffin and cut into 4 μm-thick sections. After evaporating the paraffin at 60°C for 60 min, the tissue sections were subjected to antigen retrieval by incubation with Tris-EDTA buffers. Then the endogenous peroxidase activity was blocked by hydrogen peroxide. Subsequently, the sections were stained with primary antibodies against CD3, CD4 and CD8 (Abcam), which were used at the dilutions indicated by the manufacturer. Detection of immunoreactions was performed by using the Dako-Cytomation Envision Horseradish Peroxidase System (Dako). Then the sections were counterstained with hematoxylin (Sigma).

### Statistical analysis

All results are expressed as mean ± SD. Statistical comparisons were made using a two-tailed Student’s t-test (between two groups) or a one-way analysis of variance (for multi-group comparisons). P < 0.05 was considered to represent a significant difference. Analysis and graphing were performed using the Prism 7.0 software package.

## Results

### Morphology and phenotype

**Figure 1:**
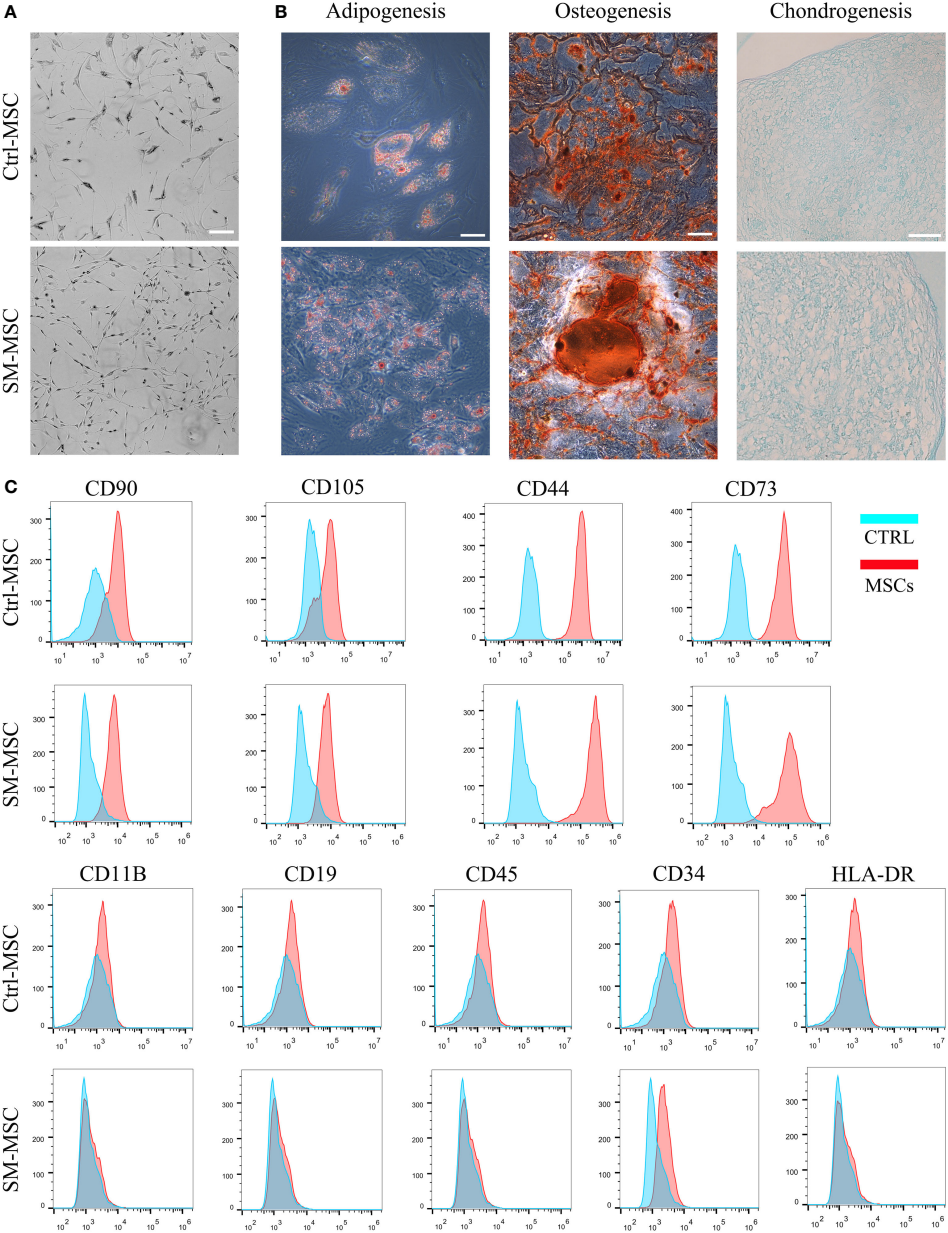
Analysis of MSC phenotype and differentiation potential. **(A)** Morphology of MSCs cultured with or without SM-ACY. **(B)** Adipogenic, osteogenic, and chondrogenic differentiation properties of MSCs. **(C)** Analysis of MSC surface marker expression. Scale bar = 100μm.

We isolated and expanded MSCs from adipose tissue and cultured the cells until passage 5. Under the condition of ACY stimulation, MSCs exhibited finer and more homogeneous morphology (**Figure 1A**). MSCs cultured with or without SM-ACY (SM-MSCs or Ctrl-MSCs) both possessed the potential of adipogenic, osteogenic and chondrogenic differentiation (**Figure 1B**). Flow cytometric analysis revealed that both kinds of MSCs were positive for typical MSC surface markers such as CD90, CD105, CD44 and CD43, while they did not express the negative MSC surface markers such as CD11B, CD19, CD45, CD34 and HLA-DR (**Figure 1C**).

### MSCs/T cells co-culture

**Figure 2:**
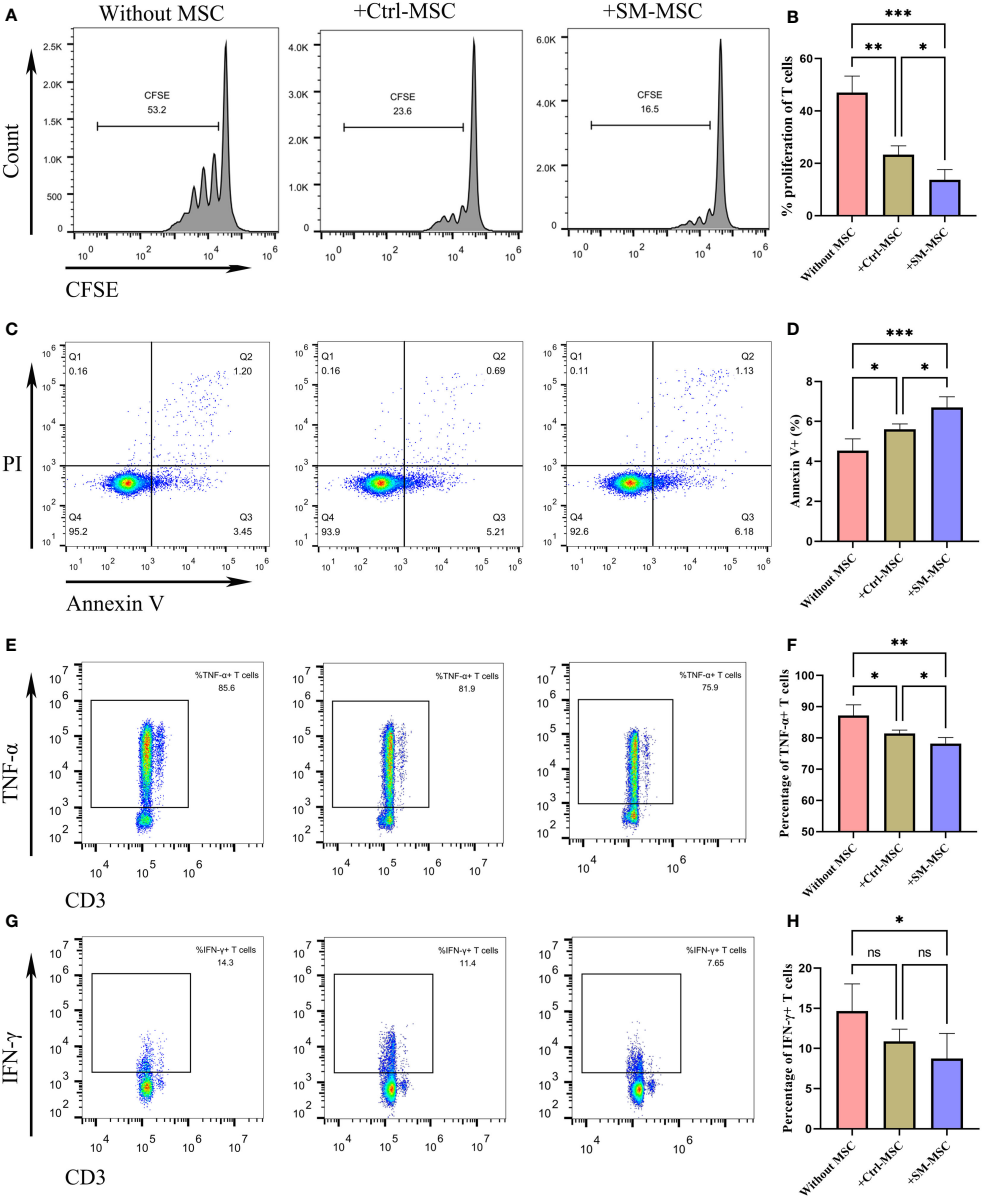
Comparison of immunosuppressive effects on activated T cells. **(A, B)** The proliferation levels of T cells were analyzed by CFSE staining, n=4 per group. **(C, D)** T cells were analyzed for apoptosis using PI & Annexin V staining, n = 5 per group. The expression levels of TNF-α **(E, F)** and IFN-γ **(G, H)** were analyzed by flow cytometry, n = 6 per group. Data are shown as mean ± SD, *P < 0.05, **P < 0.01, ***P < 0.001 and ns means not significant.

To identify whether SM-ACY contributed to the immunomodulatory properties of MSCs, we compared the proliferation rate of co-cultured activated T cells. As shown in **Figures 2A, B**, the proliferation rate of T cells co-cultured with MSCs were significantly decreased, and SM-MSCs showed a better inhibitory effect. Although the effect was mild, MSCs showed a pro-apoptotic effect on activated T cells, and more Annexin V positive T cells existed in the group of co-cultured with SM-MSCs (**Figures 2C, D**). In addition, we examined the effects of SM-ACY on the production of pro-inflammatory cytokines from activated T cells. The results showed that T cells co-cultured with MSC significantly reduced the expression of TNF-α and IFN-γ, and SM-MSCs showed a more obvious inhibition effect on these pro-inflammatory cytokines (**Figures 2E–H**).

### MSCs alleviated contact hypersensitivity induced by DNFB

**Figure 3:**
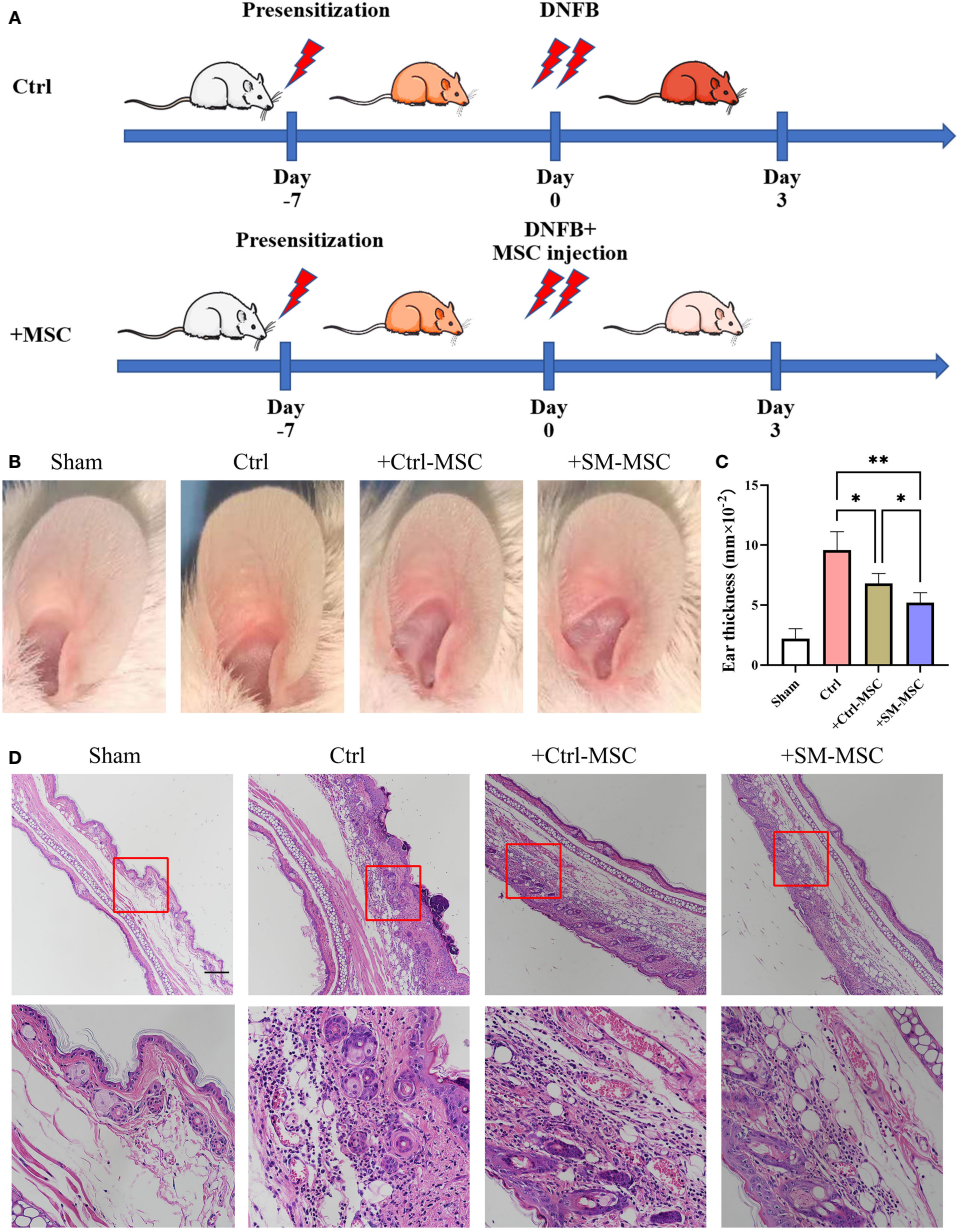
MSCs alleviate DNFB-induced contact hypersensitivity. **(A)** Schematic of experimental protocol for establishing mice contact hypersensitivity and the subsequent MSC treatment. **(B)** Representative images of ears from mice. **(C)** Ear thickness of different groups, n = 5 per group. **(D)** Histopathologic analysis was determined 2 days after injection of the cells, scale bar = 200μm. Data are shown as mean ± SD, *P < 0.05, **P < 0.01.

To evaluate the function of these two groups of MSCs in T cell-mediated immune responses *in vivo*, we induced contact hypersensitivity in mice by DNFB, with or without MSCs for alleviation, and analyzed the therapeutic effects 48h after elicitation (**Figure 3A**). The ear thickness of mice was significantly increased 48 hours after DNFB induction, while the degree of ear swelling was reduced in MSCs-treated mice. Compared with mice injected with control MSCs, SM-MSC-injected mice exhibited less swelling in the ears (**Figures 3B, C**). Hematoxylin–eosin staining also indicated that there was a difference in the status of ear swelling and inflammation cell infiltration (**Figure 3D**). Mice injected with Ctrl-MSCs or SM-MSCs showed an obvious status alleviation, and SM-MSCs exhibited stronger improvement.

### MSCs restored the infiltration of T cells in inflammatory ear tissue

**Figure 4:**
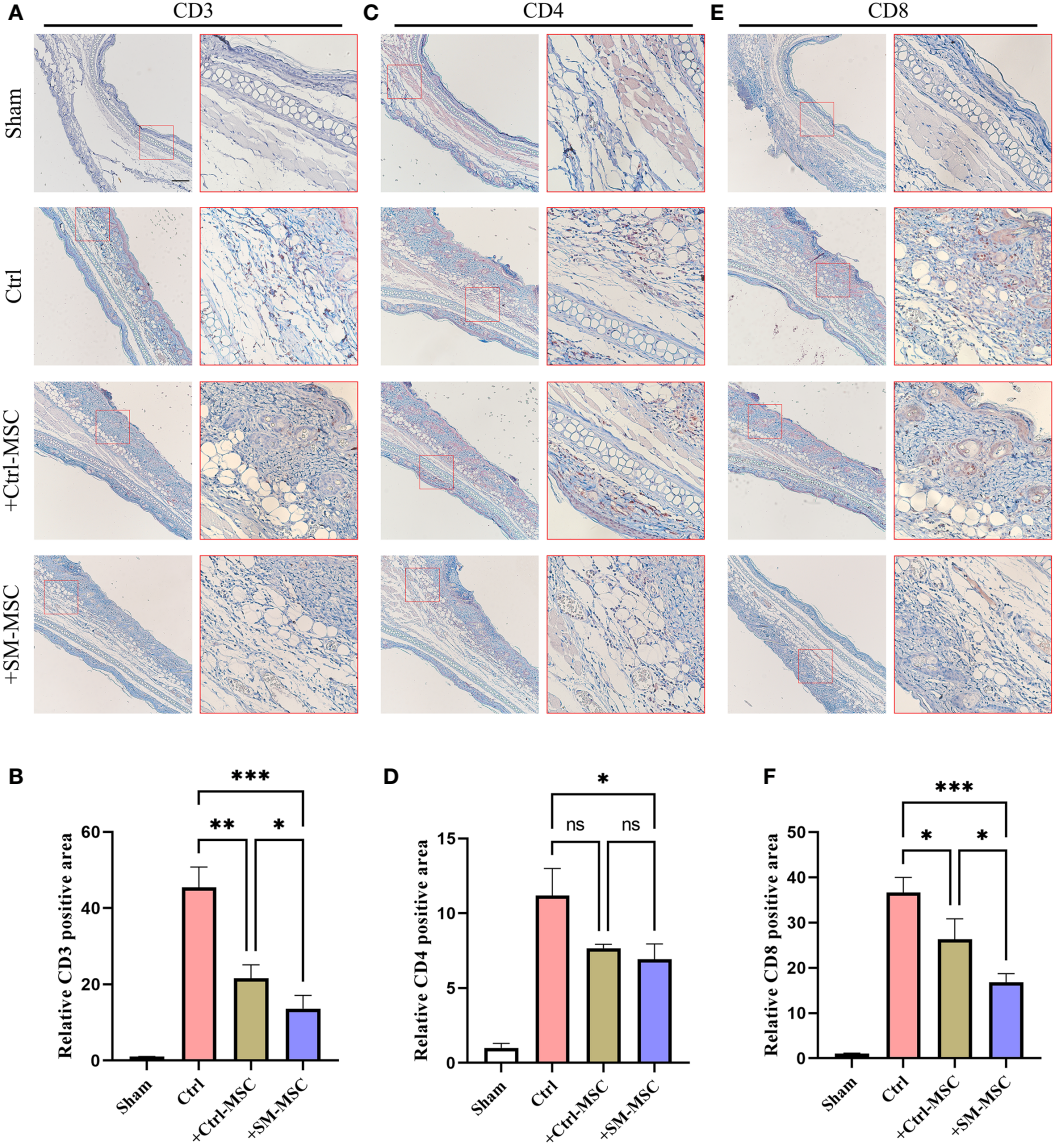
MSCs decrease T cells in ears of mouse contact hypersensitivity model. CD3 **(A, B)**, CD4 **(C, D)** and CD8 **(E, F)** positive areas of ears were calculated under fluorescence microscope, n=4 per group. The relative fluorescence positive area in the Sham group were normalized to 1.0. Scale bar = 200μm. Data are shown as mean ± SD, *P < 0.05, **P < 0.01, ***P < 0.001 and ns means not significant.

Contact hypersensitivity is mainly manifested by inflammatory T cell infiltration (26). To further investigate the MSCs-mediated inhibition of T cells in contact hypersensitivity, we analyzed the levels of the infiltration of T cells in inflammatory ear tissues. The levels of infiltration of CD3^+^ T cells were increased after DNFB induction, and MSC injection could effectively reduce the level of CD3^+^ T cells (**Figures 4A, B**). Compared with Ctrl-MSC group, the levels of infiltration of CD3^+^ T cells were lower in SM-MSCs group. The similar phenomenon was observed in the infiltration of CD4^+^ (**Figures 4C, D**) and CD8^+^ (**Figures 4E, F**) T cells, and SM-MSCs showed more power in inhibition of CD8^+^ effector T cells.

### MSCs restored the inflammatory T cells in cervical lymph nodes

**Figure 5:**
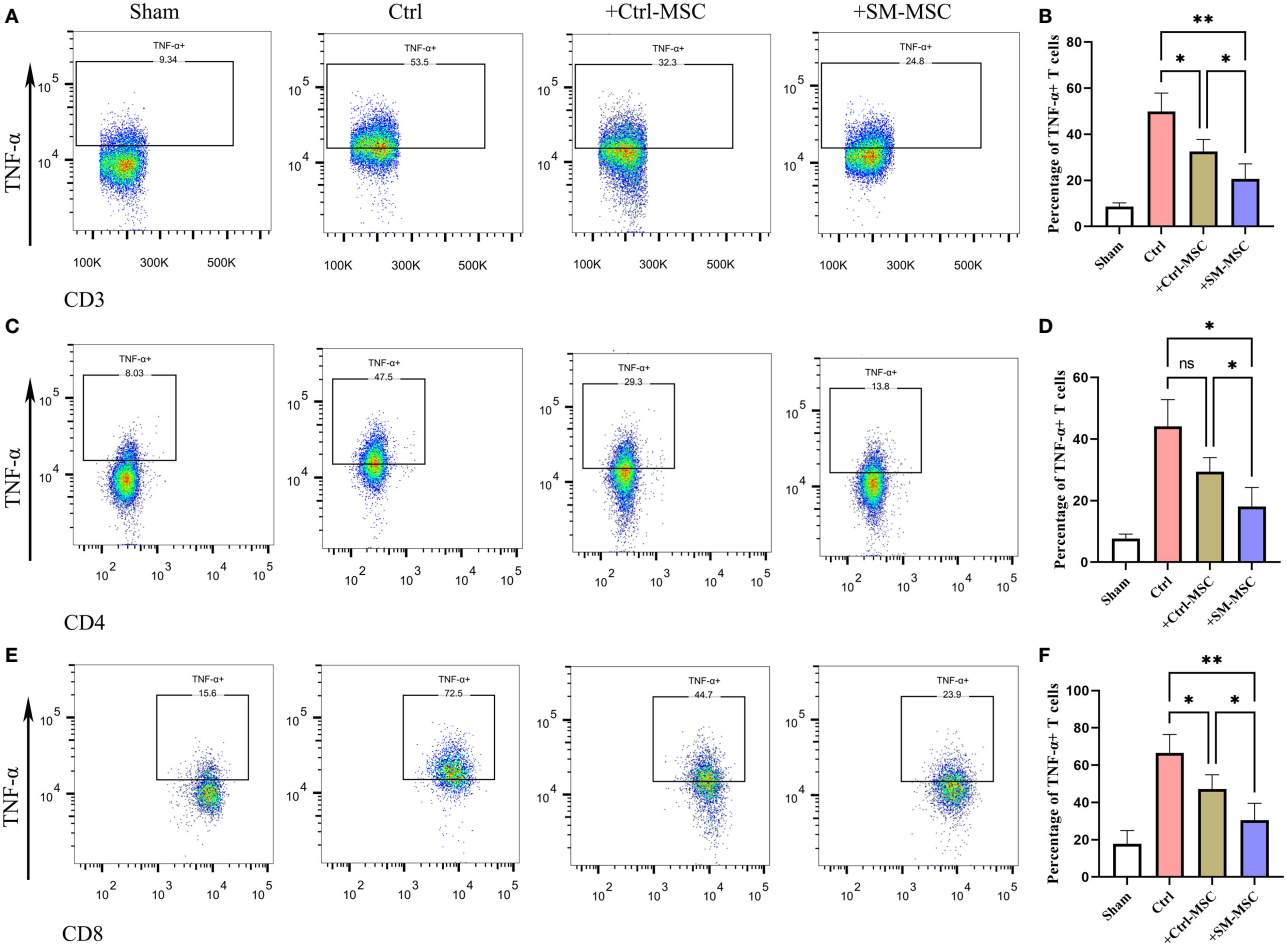
Comparison of inflammatory T cell in cervical lymph nodes. Analysis of TNF-α-producing CD3 **(A, B)**, CD4 **(C, D)** and CD8 **(E, F)** positive T cells within cervical lymph nodes, n = 5 per group. Data are shown as mean ± SD, *P < 0.05, **P < 0.01, and ns means not significant.

MSCs were found to ameliorate DNFB-induced contact hypersensitivity by reducing the response and migration of effector T cells to the draining lymph node (24, 27–29). Accordingly, we investigated whether MSCs could affect the draining lymph node cells of mice that were subjected to experimentally-induced contact hypersensitivity. As expected, the results revealed that the percentages of CD3^+^ TNF-α producing effector T cells, were remarkably increased in the cervical lymph nodes of contact hypersensitivity mice compared to the group of Sham (**Figures 5A, B**). Consistent with the *in vitro* and *in vivo* results presented above, the percentage of CD3^+^ TNF-α producing pro-inflammatory effector T cells was strongly suppressed by Ctrl-MSCs and was more effectively suppressed by SM-MSCs (**Figures 5A, B**). In addition, SM-MSCs treatment also exhibited stronger abilities to suppress CD4^+^ (**Figures 5C, D**) and CD8^+^ TNF-α producing effector T cells (**Figures 5E, F**). These results suggest that SM-MSCs might possess stronger ability to alleviate contact hypersensitivity responses by suppressing the pro-inflammatory effector T cells.

### MSCs restored the inflammatory macrophages of LPS-induced endotoxemia

**Figure 6:**
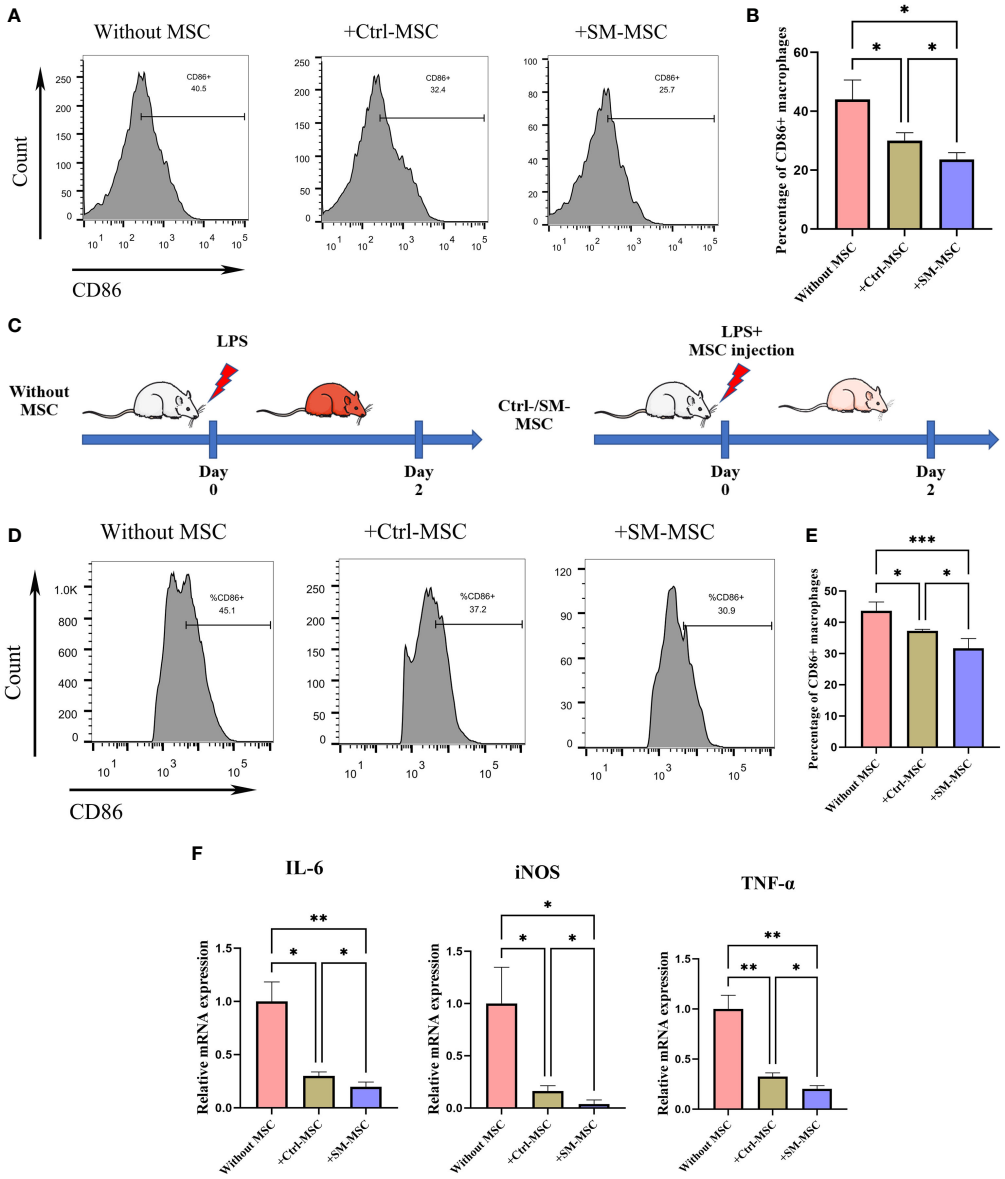
Comparison of immunosuppressive effects on activated macrophages. **(A, B)** Flow cytometry was adopted to analyze the proportion of CD86 positive M1 macrophages. n = 4 per group. **(C)** Schematic of experimental protocol for establishing LPS-induced endotoxemia model and the subsequent MSC treatment. **(D, E)** Flow cytometry was adopted to analyze the proportion of CD86 positive M1 peritoneal macrophages. n = 5 per group. **(F)** The mRNA levels of chemokines. n = 4 per group. Data are shown as mean ± SD, *P < 0.05, **P < 0.01, ***P < 0.001.

MSCs possess broad immunomodulatory functions in response to an inflammatory microenvironment which could regulate the function of macrophages (30–32). To investigate whether SM-ACY contribute to the immunomodulatory of activated macrophages, LPS-induced activated macrophages were co-cultured with two groups of MSCs. As shown in **Figures 6A, B**, LPS-activated macrophages with SM-MSCs exhibited a lower proportion of CD86 positive M1 macrophages compared to the group of Ctrl-MSCs. In *in vivo* studies, the LPS-induced endotoxemia model was used as shown in **Figure 6C**. Peritoneal macrophages from SM-MSC-treated animals had lower CD86 surface expression compared to those from without MSC-treated or with Ctrl-MSC-treated animals (**Figures 6D, E**). In addition, pro-inflammatory cytokines (TNF-α, iNOS, and IL-6) were lower in SM-MSC-treated macrophages compared to without MSC-treated or with Ctrl-MSC-treated groups (**Figure 6F**). Over all, these results demonstrated that SM-ACY could effectively improve the anti-inflammatory and immune-modulatory effects of MSCs.

## Discussion

Previous studies showed that MSCs could modulate inflammation and help tissue regeneration, which demonstrates the great potential of MSCs for the treatment of immune system diseases and regenerative medicine applications (33–35). During the expansion period of MSCs, the maintenance of their functions has always faced great challenges, and the culture environment is an important factor leading to the fluctuation of their functions (12, 36, 37). The aim of this study was to find a method to improve the immunomodulatory function of MSCs. The main goal of this technology was to allow cells possess stronger immune response properties, enhancing the immunosuppressive effect on immune cells such as activated T cells and macrophages.

Since the application value of MSCs lies in clinical treatment, the safety of the method is particularly important when developing improved technologies. Several studies have been published in which MSCs have been genetically modified based on viral transfection techniques (38–40). Although these methods can directly transform MSCs, the off-target effects associated with genetic modification and the safety of residual vectors such as viruses still pose a major obstacle to clinical transformation. Therefore, we are more inclined to explore some pretreatment techniques to improve the function of MSCs, in order to maintain the stability of MSC phenotype and avoid the introduction of inseparable components.

Numerous studies reported effective pretreatment improvement techniques. Hypoxic preconditioning was found to result in facilitated release of several trophic factors and increased immunomodulatory effect (41–43). Many studies reported the optimal combination of cytokines could improve the therapeutic effect of MSC, such as TNF-α and IFN-γ (44), IL-10 (45), HGF (46), or IDO (47). Our previous study also found that a cocktail of small-molecule compounds SM-ACY could improve the growth of MSCs (23). Moreover, we further discovered that SM-ACY which involving in three different signaling pathways respectively were capable to enhance the immunoregulatory effects of MSCs *in vitro/vivo*. However, SM-ACY did not act like a stimulator that can directly stimulate the release of functional cytokines from MSCs, but was used as a long-term supplementary medium in the early stage of MSC culturing.

A-83–01 is an inhibitor of the TGF-β signaling pathway. It is reported that TGF-β1 knockdown in human umbilical cord MSCs significantly attenuated the upregulation of inflammatory cytokines and strengthened the protective effects in MSCs against subarachnoid hemorrhage in a rat model (48). However, the detailed mechanisms underlying the increased immunoregulatory effects of MSCs directly induced by TGF-β inhibition still remain unclear. As TGF-β has been long considered as a key mediator in fibrosis in multiple tissues (49–52), our present study here suggests that MSCs treated by these compounds may also play a potential therapeutic role in fibrosis.

CHIR99021 is an activator of the classic Wnt/β-catenin signaling pathway. In the inflammatory microenvironment, pro-inflammatory cytokines like IL-1β, IL−18, IFN−γ or TNF−α can suppress MSC survival, proliferation and differentiation, thus impairing the therapeutic effects of MSCs (53–56). Of note, Wnt signaling has been found to be responsible for the homeostasis, stemness, self-renewal and proliferation of multiple adult stem cell populations, including MSCs (57). Additionally, Wnt pathway can be inhibited by TGF-β activation (58). That may explain why our strategy of Wnt signaling activation combined with TGF-β signaling inhibition synergistically enhance MSC efficacy in different animal models of inflammatory diseases.

Moreover, Y-27632 is an inhibitor of the RhoA/ROCK signaling pathway. Few studies focused on the regulating effects of ROCK pathway in immunoregulatory properties of MSCs. However, it was reported that inhibition of ROCK pathway in pancreatic stellate cells showed the potent anti-inflammatory functions by significantly reducing the infiltration of macrophages and increasing the percentage of CD4+ T cells (59). These results are consistent with our findings since MSCs and stellate cells are both stromal components in inflammatory microenvironment. What’s more, inhibition of ROCK pathway could improve the proliferation of MSCs (60), therefore promoting their anti-inflammatory effects in diseased environment.

In summary, our small-molecule compounds involving in three signaling pathways might synergistically help to enhance the immunoregulatory ability of MSCs by various mechanisms.

## Data availability statement

The original contributions presented in the study are included in the article/supplementary material. Further inquiries can be directed to the corresponding authors.

## Ethics statement

The animal study was reviewed and approved by the Medical Ethics Committee of the Third Affiliated Hospital of SYSU.

## Author contributions

JC, YF, XF, and DW performed experiments, analyzed data and wrote the manuscript. XL, ZR, and CL performed experiments and analyzed data. NL, CJ, and QZ designed experiments, analyzed and interpreted data and supervised the laboratory studies. All authors contributed to the article and approved the submitted version.

## Funding

This research was financially supported by the National Natural Science Foundation of China (Grant No: 81802849, 82100560, 81760112, 81870449, 81402426), Science and Technology Program of Guangzhou (Grant No: 202102010310), Medical Scientific Research Foundation of Guangdong Province (Grant No: A2021110), Natural Science Foundation of Guangdong Province (Grant No: 2022A1515012650).

## Conflict of interest

The authors declare that the research was conducted in the absence of any commercial or financial relationships that could be construed as a potential conflict of interest.

The reviewer CW declared a shared affiliation with the authors to the handling editor at the time of the review.

## Publisher’s note

All claims expressed in this article are solely those of the authors and do not necessarily represent those of their affiliated organizations, or those of the publisher, the editors and the reviewers. Any product that may be evaluated in this article, or claim that may be made by its manufacturer, is not guaranteed or endorsed by the publisher.
